# Multimodal diagnostic approach for identifying *Actinomyces odontolyticus* pneumonia: a case report and literature review

**DOI:** 10.3389/fmed.2025.1607223

**Published:** 2025-07-02

**Authors:** Ya Shen, Xiao-Yu Cao, Jing-Feng Shi, Zi-Xiao Cao, Xiao-Bao Teng, Ming-Feng Han

**Affiliations:** Department of Respiratory and Critical Care Medicine, Fuyang Infectious Disease Clinical College of Anhui Medical University, Fuyang, Anhui, China

**Keywords:** *Actinomyces odontolyticus*, pneumonia, next-generation sequencing, pathology, case report

## Abstract

The emergence of next-generation sequencing (NGS) has significantly improved the detection rate of Actinomyces species in pulmonary actinomycosis (PA), a rare and diagnostically challenging infectious disease. Here, we report a case of an elderly patient with a history of pyoderma gangrenosum who presented with pulmonary infiltrates and was subsequently hospitalized. Both histopathological examination of tracheoscopic biopsy specimens and NGS analysis of bronchoalveolar lavage fluid (BALF) confirmed infection with *Actinomyces odontolyticus* (*A. odontolyticus*). The patient exhibited marked clinical and radiological improvement following a period of amoxicillin/clavulanic acid therapy. Additionally, we performed a retrospective analysis of 11 documented cases of *A. odontolyticus*-associated pneumonia reported between 1978 and 2025. These cases were primarily diagnosed through pathogen culture and histopathology, with most patients demonstrating favorable outcomes following timely intervention. The age distribution of the cohort ranged from 11 to 64 years, with cough, sputum production, and dyspnea being the predominant symptoms. Notably, chest imaging findings varied widely. We anticipate that these findings will enhance clinical awareness of PA, facilitating early detection, accurate diagnosis, and improved patient management. To our knowledge, this represents the first reported case of *A. odontolyticus* pneumonia diagnosed using a combined NGS and histopathology approach, as well as the largest case series published for this condition.

## 1 Introduction

Actinomyces, a Gram-positive, branching, anaerobic bacterium of the Actinobacteriaceae family, exhibits parthenogenetic reproduction and predominantly colonizes the female genital tract, gastrointestinal system, and oral mucosa (1). While infections caused by this organism commonly involve the head, neck, craniofacial structures, thoracic cavity, abdomen, and pelvis, central nervous system manifestations are rare, accounting for ~15% of cases associated with Actinomyces infections (2). The primary route of Actinomyces transmission is oral inhalation; however, predisposing factors such as inadequate oral hygiene, excessive alcohol consumption, chronic pulmonary conditions, corticosteroid use, and immunosuppressive therapies significantly elevate infection risks (2, 3). Pathogenic species within this genus include *A. meyeri, A. turicensis, A. israelii, A. neuii, and A. odontolyticus*. Among these, *A. meyeri* is the most frequently isolated, whereas *A. odontolyticus* remains less common in clinical settings (4, 5).

PA often presents without distinctive clinical manifestations such as cough, sputum production, hemoptysis, fever, or dyspnea, making it difficult to distinguish from other pulmonary infections (6). Radiological findings may include cavitary lesions, consolidations, nodular opacities, or pleural involvement, with potential complications such as pleural effusion or empyema—features that further complicate differentiation from alternative diagnoses (7). Currently, PA diagnosis primarily relies on microbial culture and histopathological examination. However, NGS has recently emerged as a valuable tool for enhancing the detection of Actinomyces infections (8). In this report, we describe a case of *A. odontolyticus*-induced pneumonia in a patient on prolonged immunosuppressive and corticosteroid therapy. The patient presented with multiple pulmonary masses, solid consolidations, fever, and productive cough. Definitive diagnosis was achieved through histopathological assessment and NGS analysis. Fortunately, early intervention resulted in successful treatment and a positive clinical outcome.

## 2 Case presentation

**Figure 1:**
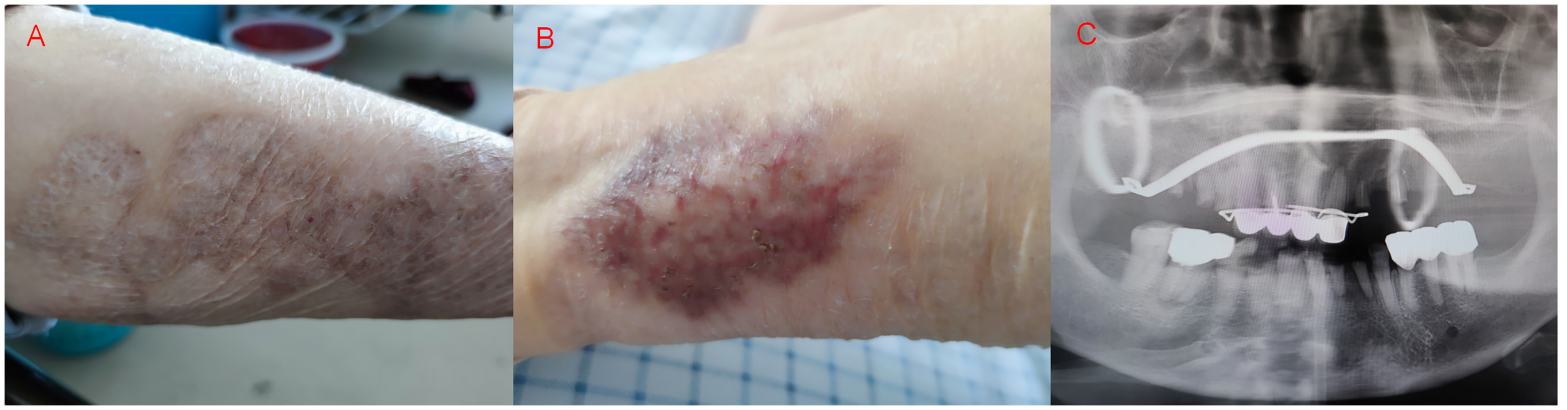
Skin damage of the patient's left lower extremity and dental plain radiographs, **(A, B)** Neovascularization, crusting, flaking, and hyperpigmentation were seen on the patient's left lower extremity skin, **(C)** Patient with multiple chipped and destroyed teeth, artificial crown and bracket placement visible.

**Figure 2:**
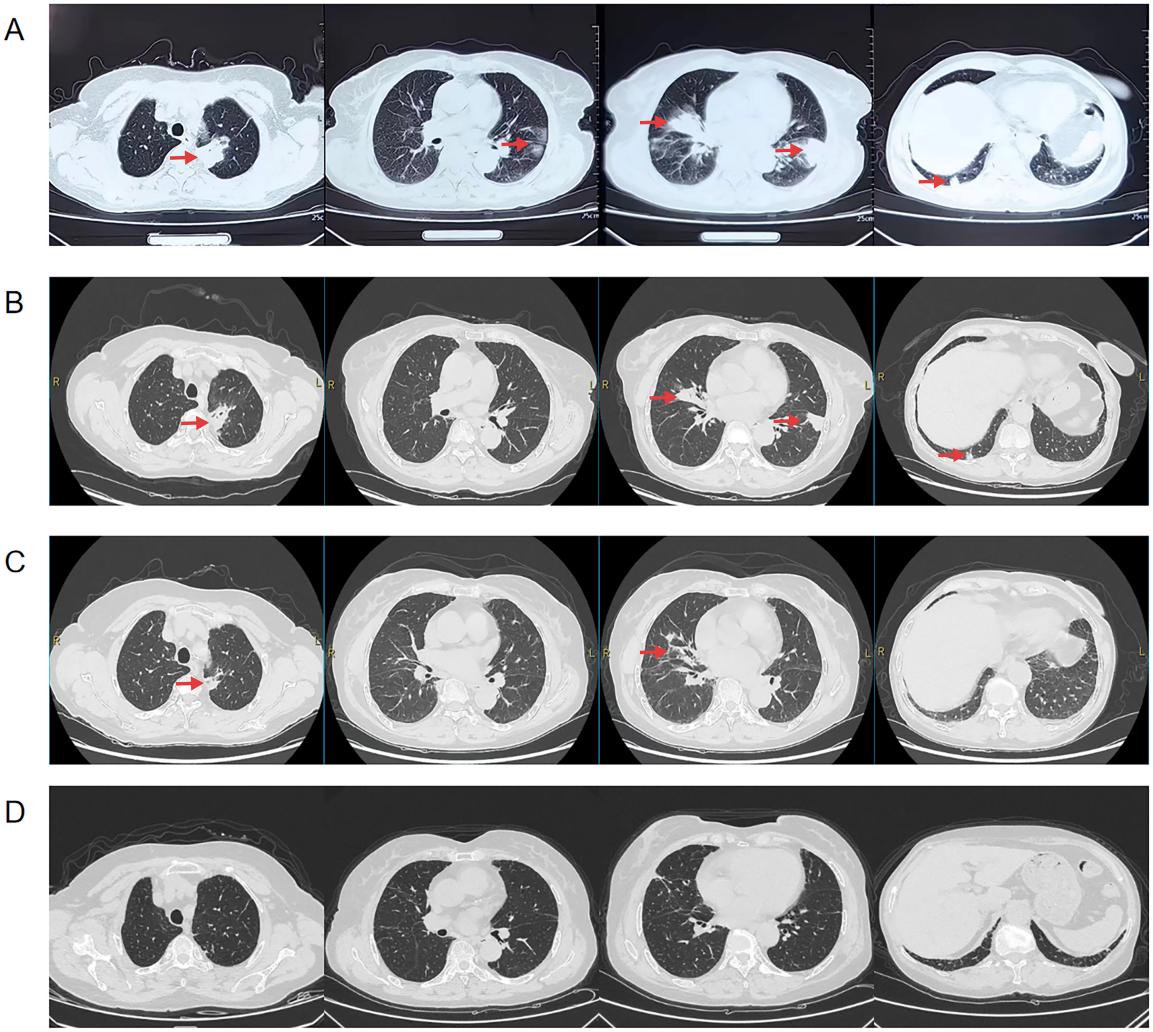
Chest CT of the patient before admission, 2 weeks of anti-infective treatment and after discharge, the location of the lesion is marked with a red arrow, **(A)** Pre-admission chest CT suggests multiple patchy, clumped, and ground-glass hyperdense shadows in both lungs, **(B)** Chest CT after 2 weeks of anti-infective treatment (amoxicillin/clavulanic) showed partial resorption of the lesion compared to the previous one, **(C)** Repeat chest CT 1 month after discharge suggests continued lesion resorption, **(D)** Chest CT 4 month after discharge suggests continued lesion resorption.

A 71-year-old woman, who has had “gangrenous pyoderma” for more than 20 years, with recurrent skin ulceration and pus on the lower limbs, was not formally treated at first but started to take “cyclosporine, and methylprednisolone” 3 years ago. Half a year ago, the skin of the left lower limb ulcerated again, and after symptomatic treatment (the specific treatment is unknown), the skin lesions have improved compared with the previous one (Figures 1A, B). In addition, the patient had a history of dental caries and tooth loss for more than 10 years, and the dental radiographs are shown in Figure 1C. The patient had no history of smoking or drinking, no tumor, and no history of chronic liver or kidney disease. This time, more than 10 days ago, cough, cough sputum, mostly paroxysmal cough, cough white sputum, and a small amount of yellow sputum, in the outside hospital chest CT suggests that: both lungs can be seen in the multiple patchy, mass-like high-density shadows (Figure 2A), to be treated with anti-infective treatment (ceftazidime 2 g q12h), the patient's symptoms did not improve significantly. Therefore, she came to our hospital for consultation. Combined with the chest imaging manifestations, the patient was admitted to the hospital for further examination, considering that the nature of the lung lesion was unknown and that there was a possibility of tumors, tuberculosis, and other lesions.

**Figure 3:**
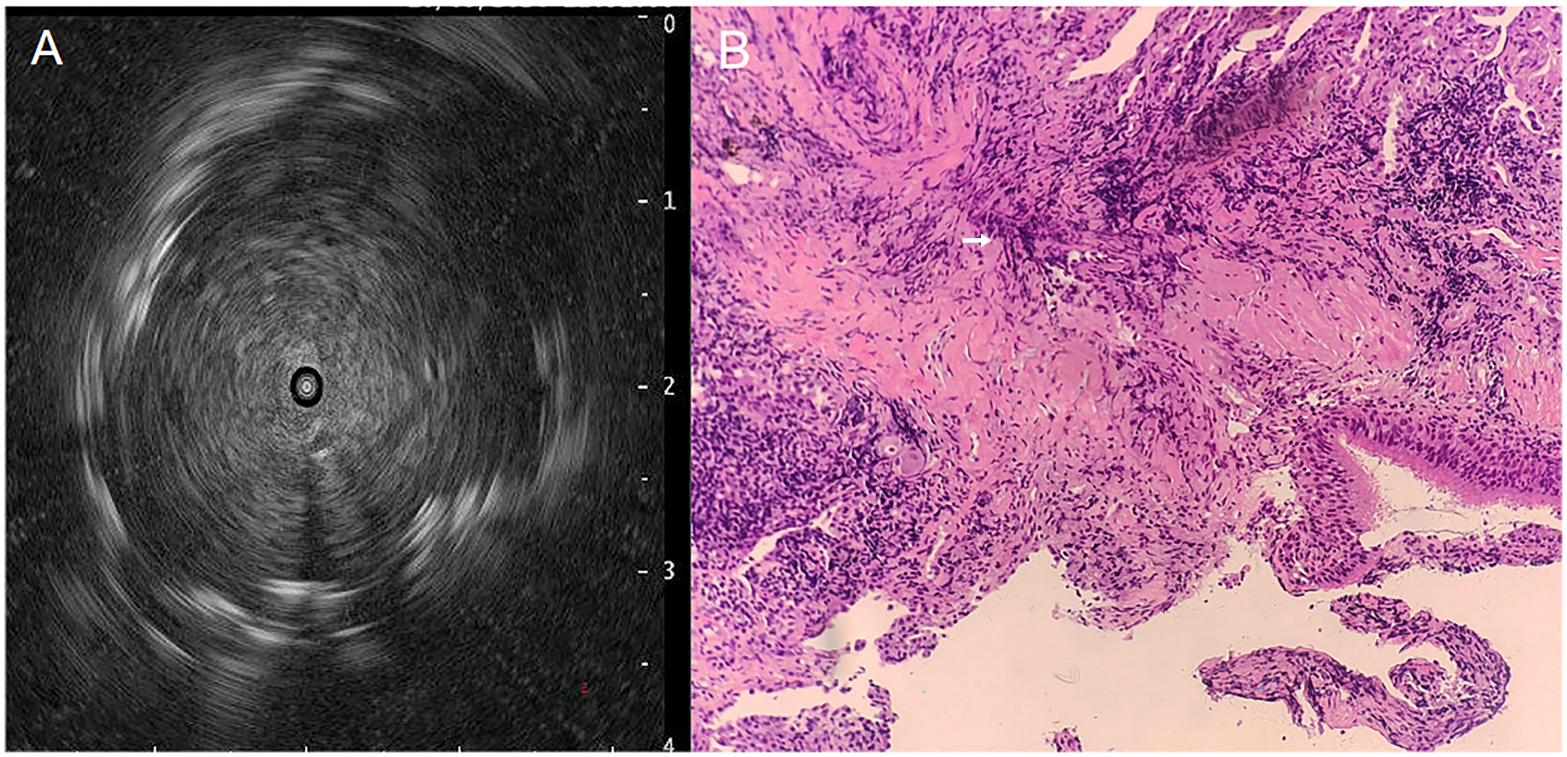
Ultrasound bronchoscopy images and PAS staining pictures, **(A)** Tracheoscopic small-ultrasound probe detects solid echoes in the apical segment of the left upper lobe, **(B)** PAS staining of tracheoscopic biopsy tissue reveals Actinomyces hyphae (200×), see white arrow markings.

Vital signs of the patient on admission: temperature 36.7°C, Pulse 86 beats/min, Blood pressure 123/64 mmHg, Respiratory Rate 16 beats/min, computed tomographic (CT) of the whole abdomen and the head did not show any obvious abnormality, laboratory tests: leukocyte count 10.92^*^10^9^/L, percentage of neutrophils 67.0%, C-reactive protein 78.6 mg/L, fungal G, GM test, lung tumor markers, T-cell test for tuberculosis infection, sputum tuberculosis-DNA, antinuclear antibody, T-lymphocyte subpopulation, cryptococcal podoconiosis antigen test, liver and kidney function, fecal routines, urinary routines did not show any abnormality. We also performed sputum and blood cultures, both of which were negative. Ultrasonic bronchoscopy suggested that the lumen of the bronchioles in each lobe segment was clear bilaterally, solid echoes were detected by a small ultrasound probe in the apical segment of the left upper lobe (Figure 3A), and biopsy was taken here for pathologic examination, while BALF was retained for NGS pathogen culture.

NGS was performed on bronchoalveolar lavage fluid (BALF) samples. Following cell lysis and centrifugation, DNA was extracted from 600 μL supernatant using the Genskey DNA Extraction Kit (1901, Genskey Biotech, Tianjin, China). Extracted DNA was ultrasonically fragmented to 200–300 bp and processed using the Genskey NGS Library Prep Kit (1906) for end repair, adapter ligation, and PCR amplification. Library quality was assessed using an Agilent 2100 Bioanalyzer (Agilent Technologies, Santa Clara, USA) and quantified by qPCR. Pooled libraries were sequenced on an Illumina NextSeq 550 instrument (SE75 mode, ≥20 M reads). Bioinformatics analysis involved filtering low-quality reads (< 70 bp) and human sequences, followed by alignment to microbial reference databases for pathogen identification.

Pathology suggests: lung tissue and bronchial mucosal tissue, long rod-shaped mycelium-like material can be seen, periodic acid-Schiff (PAS) staining suggests that the necrotic material detected long rod-shaped bacteria, morphology is considered to be actinomycetes (Figure 3B). NGS revealed 13,283 sequence reads matching *A. odontolyticus* (estimated pathogen concentration < 10^3^ copies/ml), while the BALF culture results were negative.

Following the patient's admission to the hospital, cefoperazone/sulbactam (3 g q12h) was administered as anti-infective therapy; however, the patient's cough and sputum symptoms did not significantly improve. Upon completion of pertinent examinations, taking into account the patient's history of long-term use of immunosuppressants and glucocorticoids, as well as her oral hygiene, pathology, and NGS results, the patient was diagnosed with pulmonary actinomycosis. We promptly switched the anti-infective regimen to amoxicillin/clavulanic (1.2 g q8h) to treat the patient's cough and sputum symptoms; following this modification, the patient's symptoms of cough and sputum improved. No adverse reactions occurred during the patient's use of the drug. Two weeks later, a second evaluation of chest CT revealed that the lung lesions had partially improved compared with the previous one (Figure 2B), and she was discharged from the hospital. After discharge, it was recommended to continue oral treatment with amoxicillin/clavulanic (0.914 g q12h), and chest CT after 1 month revealed that the lung lesions continued to be absorbed compared with the previous one (Figure 2C). The patient was told to return to the hospital for a follow-up in 3 months and is currently receiving regular oral amoxicillin/clavulanic potassium medication. The patient came to the hospital 3 months later for a follow-up chest CT, which indicated that the lesion was basically completely absorbed (Figure 2D), and there were no obvious abnormalities in blood counts, liver and kidney functions. According to the guideline recommendations for pulmonary actinomycosis (9), which require a 6–12 month course of anti-infective therapy, considering the patient's long-term application of glucocorticoids and immunosuppressants, and in order to reduce the risk of recurrence, it is recommended that the patient continues to take amoxicillin/clavulanic potassium treatment regularly. In December 2024 the patient had another follow-up chest CT, which suggested stable lung lesions, and is still taking amoxicillin/clavulanic. The patient's entire course of diagnosis and treatment is shown in Supplementary Figure 1.

## 3 Discussion

*A. odontolyticus* was first isolated from the oral cavity by Professor Batty in 1958 (10). Subsequent clinical observations have revealed its pathogenic potential in diverse infections, affecting multiple anatomical sites including the meninges, peritoneum, pericardium, pulmonary and pleural spaces, vertebral column, uterine cervix, biliary system, and bloodstream (11, 12). As anaerobic microorganisms, Actinomycetes present significant diagnostic challenges due to their fastidious growth requirements and prolonged incubation periods, resulting in low culture positivity rates (3). While histopathological examination typically focuses on identifying characteristic filamentous structures and sulfur granules for diagnosis, certain species such as *A. odontolyticus* fail to produce these distinctive sulfur granules, further complicating the diagnostic process (13).

Recent advances in diagnostic methodologies, particularly the implementation of next-generation sequencing (NGS) and endobronchial ultrasound-guided techniques, have significantly improved the detection rates of *A. odontolyticus* infections in pulmonary and thoracic cases in contemporary clinical practice. This case report describes an elderly female patient presenting with pulmonary infiltrates, who was admitted to the hospital for further evaluation and management. In this case, the prolonged use of immunosuppressive agents and glucocorticoids, combined with deteriorating oral hygiene (dental caries and tooth loss), established a high-risk microenvironment for pulmonary colonization of *A. odontolyticus*. Pathological staining of endobronchial ultrasound-guided biopsy specimens, complemented by NGS, facilitated rapid pathogen identification. *A. odontolyticus* is an oral colonizing bacteria, so it is necessary to make a comprehensive judgment based on the patient's history, examination results, and so on, and the purely positive NGS test is insufficient to confirm the diagnosis of *A. odontolyticus* pneumonia, and the combination of pathology test and NGS can basically be a clear diagnosis. Therefore, this is the first case in which NGS combined with pathology has been used for diagnosis. This case highlights the importance of considering oropharyngeal commensal bacteria as potential pathogens in immunocompromised hosts presenting with pulmonary infiltrates. NGS plays a pivotal role in the prompt detection of anaerobic infections.

To enhance the comprehension of *A. odontolyticus* pneumonia, we conducted a systematic literature review using the PubMed database, employing the search terms: “*A. odontolyticus*, pneumonia, lung, thorax, pulmonary” for articles published from 1978 to 2025. Cases involving mixed infections or extrapulmonary manifestations were excluded. After screening, 11 articles describing 11 patients with confirmed *A. odontolyticus* pneumonia were deemed eligible. Combined with the present case, clinical data from 12 patients were analyzed (Table 1). With a mean age of 48.75 years, there were 5 male patients (41.7%) and 7 female patients (58.3%) among the 12 patients. Eight patients (66.7%) had comorbid conditions with various illnesses, they included 3 patients with malignant tumors, two patients with chronic respiratory conditions, and other patients with rheumatoid immune system disease, diabetes mellitus, chronic alcoholic liver disease, and severe skin disease. Chest CT findings revealed heterogeneous radiological presentations: solid/mass-like opacities (*n* = 5) and ground-glass opacities (*n* = 3), along with additional features such as cavitary lesions, pleural effusions, mediastinal lymphadenopathy, and pulmonary nodules. Diagnostic confirmation relied on histopathology, NGS, and microbiological culture. All patients received penicillin-based therapy (as monotherapy or in combination), with one undergoing unilateral pneumonectomy. Following treatment, clinical improvement was observed in 10 cases, while 2 patients succumbed to complications.

**Table 1:** Clinical data of 12 patients with pneumonia due to *A. odontolyticus*.

**Patients**	**Age (y)/genders**	**Comorbidity**	**Clinical manifestation**	**Imaging**	**Diagnostic method**	**Treatment programme**	**Outcome**
1 Gray and Do (14)	11/F	None	Chronic cough	Cystic bronchiectasis and ground-glass nodular opacities	Endotracheal culture	Penicillin V and tracheoscopic resection of lesions	Improved
2 Baron et al. (15)	61/F	Rheumatological immune disease	Chest pain, breathlessness, and fatigue	Lung nodules and pleural effusion	Lung tissue culture	Penicillin	Symptoms disappear and lung lesions are completely absorbed
3 Verrot et al. (16)	52/F	Bronchiectasis	Fever, cough, and sputum	Diffuse infiltrative lesions, and cavernous lesions	Sputum culture	Imipenem and Minocycline	Symptoms resolved, complete absorption of lung lesions
4 Iancu et al. (17)	37/F	Diffuse large cell lymphoma	Fever, hemoptysis, and fatigue	Posterior hilar mass and lobulated nodule	Lung tissue culture	Penicillin and Metronidazole, left lung resection	Dead
5 Ni et al. (18)	64/F	Ovarian cancer and hypertension	Fever and chest tightness	Diffuse ground-glass shadow	Alveolar lavage fluid NGS	Penicillin G	Symptomatic improvement with significant resorption of lung lesions
6 Ruan et al. (19)	63/M	None	Chest pain, cough, and sputum	Multiple pulmonary abscesses formation and pleural empyema	Pleural pus culture	Penicillin	Dead
7 Matsumoto et al. (20)	60/M	Lung squamous cell carcinoma	Asymptomatic	A mass in the left hilum and mediastinal lymphadenopathy	Alveolar lavage fluid culture and MALDI-TOF MS	Ampicillin sequential Amoxicillin	Partial resorption of lung lesions
8 Erro Iribarren et al. (21)	43/F	Asthma and chronic eosinophilic pneumonia	Catarrh and dyspnea	Pulmonary infiltrates and a small left pleural effusion	Endotracheal tissue culture	Ceftriaxone sequential Amoxicillin	Symptoms improve
9 Wang et al. (22)	34/M	None	Cough and sputum	Consolidation of cavities, exudate from surrounding capillaries, and stripy shadows	Sputum culture	Penicillin and Ornidazole	Partial resorption of lung lesions
10 Massey and Barney (23)	33/M	None	Fatigue, weight loss, night sweats, and shortness of breath	Ground-glass opacifications	Lung tissue culture	Amoxicillin	Symptoms improved with partial resorption of lung lesions
11 Vilar da Mota et al. (24)	56/M	Diabetes mellitus/chronic alcoholic liver disease	Fever, mild dyspnea, sputum, profuse nocturnal sweating, anorexia, and weight loss	Consolidation in the right lung, with multiple nodular opacities visible, pleural effusion	Blood culture	Ceftriaxone sequential amoxicillin	Symptoms improved with partial resorption of lung lesions
12 ^Case^	71/F	Gangrenous pustulosis	Cough, sputum, and no appetite	Patchy clumpy high-density shadows and lymphadenopathy	Endotracheal histopathology and alveolar lavage fluid NGS	Potassium amoxicillin clavulanate	Symptoms improved with partial resorption of lung lesions

M, Male; F, Female; NGS, next-generation sequencing; MALDI-TOF MS, matrix-assisted laser desorption/ionization time-of-flight mass spectrometry.

Current literature on PA remains limited by small case numbers. Previous studies have identified key epidemiological characteristics, including a predilection for patients aged ≥50 years, male predominance, and established risk factors such as poor oral hygiene, chronic alcohol use, and facial trauma (6, 25). Our cohort demonstrated comparable demographic features with a mean age of 48.75 years, though showed a reversed gender distribution (58.3% female). This discrepancy may reflect study limitations including small sample size and our strict inclusion criteria of pure *A. odontolyticus* pulmonary infections. While pre-existing pulmonary disease has been recognized as a risk factor for PA (3), the potential role of immunosuppression in pathogenesis remains poorly characterized due to insufficient data. In our reported case, prolonged immunosuppressive therapy and glucocorticoid administration may have contributed to disease development, mirroring observations in pyoderma gangrenosum. However, definitive conclusions await future systematic investigations of this potential association.

Hemostasis and chest discomfort were uncommon, and one patient had no discernible clinical symptoms. The most frequent clinical symptoms of the patients in this research were cough, sputum, fever, exhaustion, and dyspnea. When compared to other studies in the literature, our patients experienced a reduced incidence of hemoptysis—just one case, a higher incidence of fatigue (25%), and the same incidence of the remaining symptoms (26, 27). In addition to the above-described localized pulmonary solid lesions and mass shadow with or without intra-lesional hypodense shadow, other common symptoms on chest imaging were enlarged mediastinal lymph nodes, nodules, pleural effusion, and ground glass shadow (28). PA has occasionally been mistakenly identified as lung cancer in clinical situations due to the non-specificity of the imaging findings (29). According to Kim's study, 42 individuals had diagnoses of different infectious lung diseases, while 33 out of 94 patients had their initial diagnosis of lung cancer (6). The patients in this report had imaging presentations that were largely in line with the results of earlier research, indicating that caution needs to be used during the initial evaluation of these patients to distinguish them from malignancies and other infectious diseases of the lungs.

The definitive diagnosis of PA relies on microbiological and histopathological examination. Conventional diagnostic modalities include microbial culture, 16S ribosomal RNA (rRNA) gene sequencing, matrix-assisted laser desorption/ionization time-of-flight mass spectrometry (MALDI-TOF), and NGS, as shown in Supplementary Table 1. Histopathological analysis typically demonstrates characteristic sulfur granules and gram-positive filamentous bacilli within lesional tissue (3). Among our cohort of 12 patients, *A. odontolyticus* was identified via conventional culture in 10 cases (83.3%), while NGS and MALDI-TOF confirmed the diagnosis in **two** and **one** patient, respectively. Notably, in the presented case, *A. odontolyticus* was undetectable by culture but was successfully identified through mNGS of bronchoalveolar lavage fluid (BALF) alongside histopathological findings. Endobronchial ultrasonography-guided sheath (EBUS-GS) sampling proved invaluable in securing adequate specimens. Compared with traditional culture and pathology, NGS offers rapid (24–48 h) and comprehensive pathogen detection, significantly aiding clinical decision-making (30). Nevertheless, interpretation of NGS results requires caution, as false positives may arise due to microbial colonization or contamination. Furthermore, actinomycete cultures remain challenging, with low yield rates. Consequently, histopathological confirmation remains crucial, and a combined approach integrating NGS with pathology is emerging as a robust diagnostic strategy for PA.

Surgery and medication are used to treat PA, among the drugs used include tetracyclines, penicillins, cephalosporins, carbapenems, lincosamides, and glycopeptides. In clinical practice, penicillins are the most often used medications and are advised as the **first** option. Alternative diagnoses should be actively sought for patients who relapse or do not respond to early therapy. If necessary, the antibiotic regimen should be readjusted. Surgical treatment may be undertaken if necessary for certain individuals who have chronic hemoptysis, significant tissue necrosis, abscesses, and fistulas, or for those in whom cancer cannot be ruled out (3, 6, 23, 26). **Ten** of the patients in this paper initially selected penicillin as monotherapy or combination therapy. **One** patient initially opted for penicillin and metronidazole, but continued to develop high fever and hemoptysis, which led to a left pneumonectomy. Regrettably, the patient's condition did not improve and he passed away.

With the right care, the majority of PA patients recover completely and have a high overall cure rate. Patients who experience treatment failure or recurrence, however, could need more involved treatment plans and ongoing care. Of the 12 patients we summarized, **ten** demonstrated a progressive recovery following treatment, **one** died later from uncontrolled hemoptysis and persistent hyperthermia, and **one** died from respiratory failure brought on by severe sepsis.

## 4 Conclusion

Pneumonia caused by *A. odontolyticus* is rarely documented in medical literature. The disease presents with non-specific clinical symptoms and imaging findings, leading to frequent misdiagnosis. Current diagnostic approaches rely heavily on microbial culture and histopathological examination, though emerging technologies such as next-generation sequencing (NGS) may revolutionize future diagnostic strategies by enabling co-pathogen detection and improved microbial identification. Therapeutic options for *A. odontolyticus* pneumonia include antimicrobial therapy and surgical intervention. In cases where medical management fails or severe complications develop, early surgical intervention can be beneficial. Given the risk of recurrence, long-term clinical follow-up is essential to monitor disease progression and assess therapeutic response. While the prognosis of actinomycosis is generally favorable, treatment strategies should be tailored to individual patient factors. Close monitoring is critical, and treatment regimens should be promptly adjusted based on clinical evolution.

## Data Availability

The original contributions presented in the study are included in the article/Supplementary material, further inquiries can be directed to the corresponding authors.
