# Correction: Multimodal diagnostic approach for identifying *Actinomyces odontolyticus* pneumonia: a case report and literature review

**DOI:** 10.3389/fmed.2025.1659435

**Published:** 2025-07-18

**Authors:** Ya Shen, Xiao-Yu Cao, Jing-Feng Shi, Zi-Xiao Cao, Xiao-Bao Teng, Ming-Feng Han

**Affiliations:** Department of Respiratory and Critical Care Medicine, Fuyang Infectious Disease Clinical College of Anhui Medical University, Fuyang, Anhui, China

**Keywords:** *Actinomyces odontolyticus*, pneumonia, next-generation sequencing, pathology, case report

Authors Ya Shen, Xiao-Yu Cao, and Jing-Feng Shi were erroneously omitted as equal contributing first authors.

The original version of this article has been updated.

