# Supplementary material for: Multimodal diagnostic approach for identifying Actinomyces odontolyticus pneumonia: a case report and literature review
Source: Front Med (Lausanne). 2025 Jul 2;12:1607223. doi: 10.3389/fmed.2025.1607223 (PMC12263356; doi:10.3389/fmed.2025.1607223)
Supplement: Supplementary file 1 [file Image_1.pdf]

Supplementary Material

## Supplementary Figures

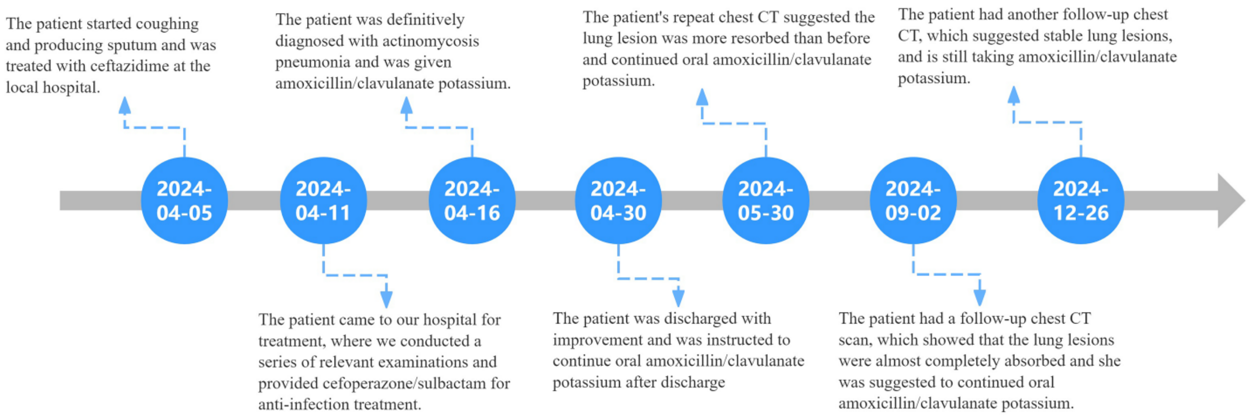


**Supplementary Figure 1.** Patient's time line from onset to diagnosis and treatment.
