# Supplementary material for: Multimodal diagnostic approach for identifying Actinomyces odontolyticus pneumonia: a case report and literature review
Source: Front Med (Lausanne). 2025 Jul 2;12:1607223. doi: 10.3389/fmed.2025.1607223 (PMC12263356; doi:10.3389/fmed.2025.1607223)
Supplement: Supplementary file 2 [file Table_1.docx]

| **Method** | **Principle** | **Advantages** | **Disadvantages** | **Typical Use Cases** |
| --- | --- | --- | --- | --- |
| Smear Microscopy | Direct staining and visualization | Fast (<1h), cheap | Low sensitivity (~10⁴ CFU/mL), subjective | Tuberculosis, malaria screening |
| Culture | Grow pathogens and biochemical tests | Gold standard, allows antibiotic testing | Slow (2–5 days), low yield (<30%) | Bacterial/fungal infections |
| PCR/qPCR | Amplifies pathogen DNA/RNA | Fast (2–4h), sensitive | Targets only known pathogens | COVID-19, flu |
| NGS | Sequences all genetic material in a sample | Detects unknown pathogens, broad, fast | Expensive, complex | Severe/unexplained infections |
| Antigen Testing | Detects pathogen surface proteins | Very fast (15–30 min), portable | Moderate sensitivity (~10³ CFU/mL) | Rapid strep/ flu/ COVID tests |
| Antibody Testing | Detects host antibodies | Retrospective diagnosis | Delayed positivity (5–7 days) | HIV, hepatitis, past infections |

**Supplementary TABLE 1** Comparison of pathogen detection methods

TB: Tuberculosis; PCR: Photo-conductive Relay; qPCR: Quantitative Photo-conductive Relay; DNA: deoxyribonucleic acid; RNA: ribonucleic acid; HIV:Human Immunodeficiency Virus.
